# The Impact of Comorbid Depression on Educational Inequality in Survival after Acute Coronary Syndrome in a Cohort of 83 062 Patients and a Matched Reference Population

**DOI:** 10.1371/journal.pone.0141598

**Published:** 2015-10-29

**Authors:** Merete Osler, Eva Prescott, Ida Kim Wium-Andersen, Else Helene Ibfelt, Martin Balslev Jørgensen, Per Kragh Andersen, Terese Sara Høj Jørgensen, Marie Kim Wium-Andersen, Solvej Mårtensson

**Affiliations:** 1 Research Center for Prevention and Health, Rigshospitalet – Glostrup, Copenhagen University, Glostrup, Denmark; 2 Department of Cardiology Y, Bispebjerg Hospital, Copenhagen, Denmark; 3 Department of Psychiatry O, Rigshospitalet, Copenhagen, Denmark; 4 Department of Biostatistics, University of Copenhagen, Copenhagen, Denmark; 5 Department of Psychiatry, Frederiksberg Hospital, Frederiksberg, Denmark; Heinrich-Heine University, Faculty of Medicine, GERMANY

## Abstract

**Background:**

Patients with low socioeconomic position have higher rates of mortality after diagnosis of acute coronary syndrome (ACS), but little is known about the mechanisms behind this social inequality. The aim of the present study was to examine whether any educational inequality in survival after ACS was influenced by comorbid conditions including depression.

**Methods:**

From 2001 to 2009 all first-time ACS patients were identified in the Danish National Patient Registry. This cohort of 83 062 ACS patients and a matched reference population were followed for incident depression and mortality until December 2012 by linkage to person, patients and prescription registries. Educational status was defined at study entry and the impact of potential confounders and mediators (age, gender, cohabitation status, somatic comorbidity and depression) on the relation between education and mortality were identified by drawing a directed acyclic graph and analysed using multiple Cox regression analyses.

**Findings:**

During follow-up, 29 583(35.6%) of ACS patients and 19 105(22.9%) of the reference population died. Cox regression analyses showed an increased mortality in the lowest educated compared to those with high education in both ACS patients and the reference population. Adjustment for previous and incident depression or other covariables only attenuated the relations slightly. This pattern of associations was seen for mortality after 30 days, 1 year and during total follow-up.

**Conclusion:**

In this study the relative excess mortality rate in lower educated ACS patients was comparable with the excess risk associated with low education in the background population. This educational inequality in survival remained after adjustment for somatic comorbidity and depression.

## Introduction

A large number of studies reviewed in Table 1 [1–22] have shown that patients with low socioeconomic position (SEP) have higher rates of mortality after diagnosis of acute coronary syndrome (ACS). Furthermore, some of these studies have examined factors that might explain this social inequality [1,5–7,9–11,14,15,18–22]. ACS patients with low SEP more often have ACS risk factors [7,10,20], more severe ACS and somatic comorbidity [1,4,5,7,9,10,14,15,18,20–22], wait longer for acute treatments [2,6,7,22, 23] and receive less advanced treatments [1,2,5–7,9,12,19–21, 24,25]. However, in most studies adjustment for ACS risk factors and severity of the disease, somatic comorbidity, waiting time and treatment factors only seem to explain a part of the social inequality in survival after ACS [1,6,7,9–11,14,15,18–22]. Depression is more frequent in groups with lower SEP and is also a significant prognostic factor for ACS [26,27] and might thus contribute to explain the social inequality in survival after ACS [28] but this is scarcely investigated. As Table 1 shows, 12 of the studies listed in Table 1[1,4–7,9,10,14,15,19,21,22] have examined the influence of comorbidity on the association between SEP and survival after ACS, but they have only accounted for somatic comorbidity, but not examined the influence of depression. Further, none of the studies have included a reference population to compare whether the social inequality in mortality in ACS patients compares to the mortality inequality in the general population. A difference between ACS patients and the reference population might reflect the influence of factors attributed to the disease (ACS). The aims of the present study were 1: to examine whether educational status was associated with mortality in first time ACS patients compared to a matched reference population and 2: to explore if any educational differences in mortality were explained by comorbid conditions including depression.

**Table 1:** Studies exploring the association between socioeconomic position (SEP) and survival after incident Acute Coronary Syndrome (ACS).

Author^reference^ (year of publication)	Study population	Country and Study period	Measure of SEP (income, education or occupation)	Length of follow-up for all-cause mortality	Explanatory factors (besides gender and age) adjusted for	Main finding
Alter et al. ^1^ (1999)	51 591 hospitalised AMI patients	Canada, 1994–1997	Neighborhood income	1 year	Severity of ACS, somatic comorbidity, speciality of physician	Income inequality in 1 year mortality. Slightly attenuated by adjustment
Salomaa et al.^2^ (2001)	8467 first time AMI patients	Finland, 1983–1992	Education and income	28 days and1 year		Educational and income inequality in 28 days and 1 year mortality
Cesanne et al. ^3^ (2001)	1077 AMI patients	Italy, 1993–1994	Occupational social class	28 days		Occupational inequality in 28 days mortality
Rasmussen et al. ^4^ (2006)	37 560 first time AMI patients	Denmark, 1995–2002	Education and income	30 days and median 5 years	Cohabitation status, somatic comorbidity*	Educational and income inequality in 28 day and longterm mortality
Alter et al. ^5^ (2006)	3407 AMI patients	Canada,1995–2003	Education and income	2 years	Race, cohabitation status, ACS risk factors, severity of ACS, somatic comorbidity, speciality of physician, revascularisation.	Educational or income inequality in 2 years mortality were explained by adjustment for somatic comorbidity and in hospital treatment
Bernheim et al. ^6^ (2007)	2 142 AMI patients	USA, 2003–2004	Education and income	1 year	Race, ACS risk factors, severity of ACS, somatic comorbidity, antithrombotics, revascualisation.	Educational inequality in 1 year mortality was explained by adjustment for somatic comorbidity Income inequality in 1 year mortality was slightly attenuated by adjustments
Gerber et al. ^7^ (2008)	705 AMI patients	USA, 2002	Education and Neighborhood income	Median 13 months	ACS risk factors, severity of ACS, somatic comorbidity**, antithrombotics, revascularisation.	Educational inequality in mortality was explained by adjustments. Income inequality in mortality was slightly attenuated by adjustments
Rosvall et al. ^8^ (2008)	69 223 first time AMI patients	Sweden, 1991–2003	Income	Pre hospital and 28 days		Income inequality in prehospital and 28 days mortality
Rosvall et al. ^9^ (2008)	46 407 First time AMI patients alive after 28 days	Sweden, 1993–1996	Income	5 years	Somatic comorbidity, speciality of hospital, revascularisation	Income inequality in 5 year mortality was slightly attenuated by adjustments
Gerber et al. ^10^ (2010), Molshatzky et al. ^11^ (2011)	1625 AMI patient above 64 years	Israel, 1992–1993	Neigborhood SESEducation	Median follow-up 13 years	ACS risk factors, severity of ACS,Somatic comorbidity	SES and educational inequality partly explained by adjustment.
Mehta et al. ^12^ (2011)	11 326 STEMI patients	9 countries, 1995–1997	Education	30 days and 1 year		Educational inequality in 30 days and 1 year mortality in all 9 countries.
Lammintausta et al. ^13^ (2012)	15 374 first time AMI patients	Finland, 1993–2002	SES measured by Income, Education, Occupation	28 days		SES inequality in 28 days mortality
Van Oeffelen et al. ^14^ (2012)	76 351 first time AMI patients	Netherland, 1998–2007	Income	Prehospital and 28 days	Race, cohabitation status, somatic comorbidity**	Income inequality in prehospital mortality after adjustment. Income inequality in 28 day mortality in men after adjustment.
Stirbu et al. ^15^ (2012)	15 416 first time ACS patients	Netherlands,2003–2005	Income	28 days and 1 year	Race, somatic comorbidity**	Income inequality in 28 days and 1 year mortality after adjustments
Yang et al. ^16^ (2012)	366 085 first time AMI patients	Sweden, 1987–2008	Education	28 days		Educational inequality in 28 day mortality
Foraker et al. ^17^ (2012)	9 116 first time AMI patients	USA, 1992–2002	Neighborhood income	28 days and 29–365 days		Income inequality in 28 days and 29–365 days mortality
Coady et al. ^18^ (2014)	15 972 first time AMI patients	USA, 1991–2001	Neighborhood income and Education	1 year and 1–5 years	ACS risk factors, revascualisation	Educational inequality in 1–5 year mortality in men
Gnavi et al. ^19^ (2014)	5792 first time STEMI and NSTEMIpatients	Italy, 2008	Education	30 days and 1 year	Somatic comorbidity**. hospital fascilities	Educational inequality in 30 days and 1 year mortality were explained by adjustment for age and comorbidity (but wide CI due to small number of cases)
Kirchberger et al. ^20^ (2014)	3479 first time AMIpatients	Germany 2000–2008	Education	Median 6.1 years	Cohabiation status, ACS risk factors, severity of ACS, revascularisation	Educational inequality in patients above 65 years slightly attenuated after adjustment.
Igland et al. ^21^ (2014)	111 993 first time AMI patients	Norway, 2001–2009	Education	28 days and 29–365 days	Somatic comorbidity**.	Educational inequality in 28 days and 29–365 days mortality attenuated after adjustment
Mårtensson et al. ^22^ (2015)	25 425 first time NSTEMI and unstable angina patients	Denmark, 2001–2009	Education	30 days and 1 year	Severity of ACS, somatic comorbidity*, angiography	Educational inequality in 30 days and 1 year mortality attenuated after adjustment s

STEMI,ST-elevation myocardial infarction; NSTEMI, Non-ST-elevation myocardial infarction; AMI, acute myocardial infarction. Somatic comorbidity measured following:

* the Ontarian AMI prediction rule or

** Charlson comorbidity index.

## Methods

The present study was based on linking information from the following Danish population based registers: The National Patient Register (NPR), the Civil Registration System (CPR), The Danish Prescription Register (DPR), and the Integrated Database for Labour Market Research (IDA) [29] using the unique 10 digit person identification number as key. The study was evaluated and approved by the Danish Data Protection Agency. Informed consent was not obtained as patient information was anonymized and de-identified prior to analysis. The anonymous data files are available at a server in Statistics Denmark.

### Study cohort

ACS patients were derived from the NPR, which provides information on date of admission and discharge diagnosis (according to International Classification of Disease (ICD)-10) of all diseases leading to hospital admission and outpatient’s visits by citizens in Denmark since 1979 and 1995, respectively. For this study, all first time hospitalizations of ACS in subjects over 15 years of age occurring from 1 January 2001 to 31 December 2009 were identified (n = 97 793) by the following specific ICD10 codes: I20.0 Unstable angina pectoris, I21.0-I21.3 ST-elevation myocardial infarction (STEMI), I21.4 Non-ST-elevation myocardial infarction (NSTEMI) and I21.9 an acute myocardial infarction non specified (AMI). A comparable reference population was established by 1:1 matching on gender, age, and municipality on time of ACS diagnose using information from the CPR. A reference person might later develop ACS and become a case, while an ACS case could not be sampled as reference.

### Data

#### Socioeconomic position

Information on SEP was obtained from the IDA database which holds individual level information on socioeconomic variables such as education, income and attachment to the labour marked since 1980. For the present study education was defined as the highest level of education registered at study entry and grouped in three categories: Basic education (7–9 grade of obligatory schooling), medium education (high school degree or vocational), higher education (more than high school degree). Information on educational status was not available for those born before 1921 (n = 25 010) and for 4 098 other subjects, e.g. adult immigrants. The 14 731 ACS patients and 14 377 of the reference population with missing information were excluded from the present analyses.

#### Outcome measures

The population was followed for time of death by all causes in the CPR. To investigate whether the social difference in mortality varied over time and to allow comparison with results from previous studies, deaths within 30 days and one year after study entry as well as at end of follow-up (December 2012) were main outcomes.

#### Covariables

Hospital main or secondary diagnosis of depression in both ACS patients and the reference population were obtained from the NPR using ICD-10 codes: F31.3–33;34.1. Use of antidepressant medication was obtained from the DPR which contains information on outpatient prescription drug use by all citizens of Denmark since 1995. Each prescription record contains detailed information on the drug dispensed (Anatomic Therapeutical Classification (ATC) system name), the date and the civil registration number of the person purchasing the drug. We obtained information about every prescription of antidepressants claimed by cohort members from 1995 to 2012 using the ATC codes N06A. *Previous depression* was defined as any discharge diagnosis of depression or medication with prescription of antidepressants for at least the last five years and up to fifteen years preceding the ACS event or study entry (for the reference population sample). *Incident depression* was defined as any discharge diagnosis of depression or medication with prescription of antidepressants after ACS event or study entry and the following two years. About 13% of depression cases had a ICD-10 code, while the remaining 87% were defined by an ATC-code. Antidepressants might be prescribed for various diseases and single prescriptions seem to be more frequent in subjects with no recorded depression diagnosis [30]. Consequently, we performed a sensitivity analysis excluding the 10% of cohort members who had been defined with an incident depression due to a single purchase of antidepressants. We also examined whether type of diagnosis definition (ICD-10 or ATC code) changed the influence of comorbid depression on the association between education and mortality.

Information on gender, age and cohabitation status from the CPR were also included as potential confounding or mediating factors together with data on somatic comorbidity and purchase of statins (ACT-code:C10AA) five years preceding study entry. Statin use might influence the development of depression [31] or other comorbidities and has been associated with SEP in previous studies [32]. Comorbidities were defined with data from DPR and NPR on medical treatment or hospitalization for stroke, chronic obstructive pulmonary disease, cancer, obesity, diabetes and liver-, kidney-, connective tissue and inflammatory diseases. The diseases included were assumed to be associated with both SEP and mortality. Each disease was dichotomized to yes/no and the total number of comorbidities was counted and grouped as 0; 1 to 2 and 3 or more.

### Statistical analyses

**Fig 1:**
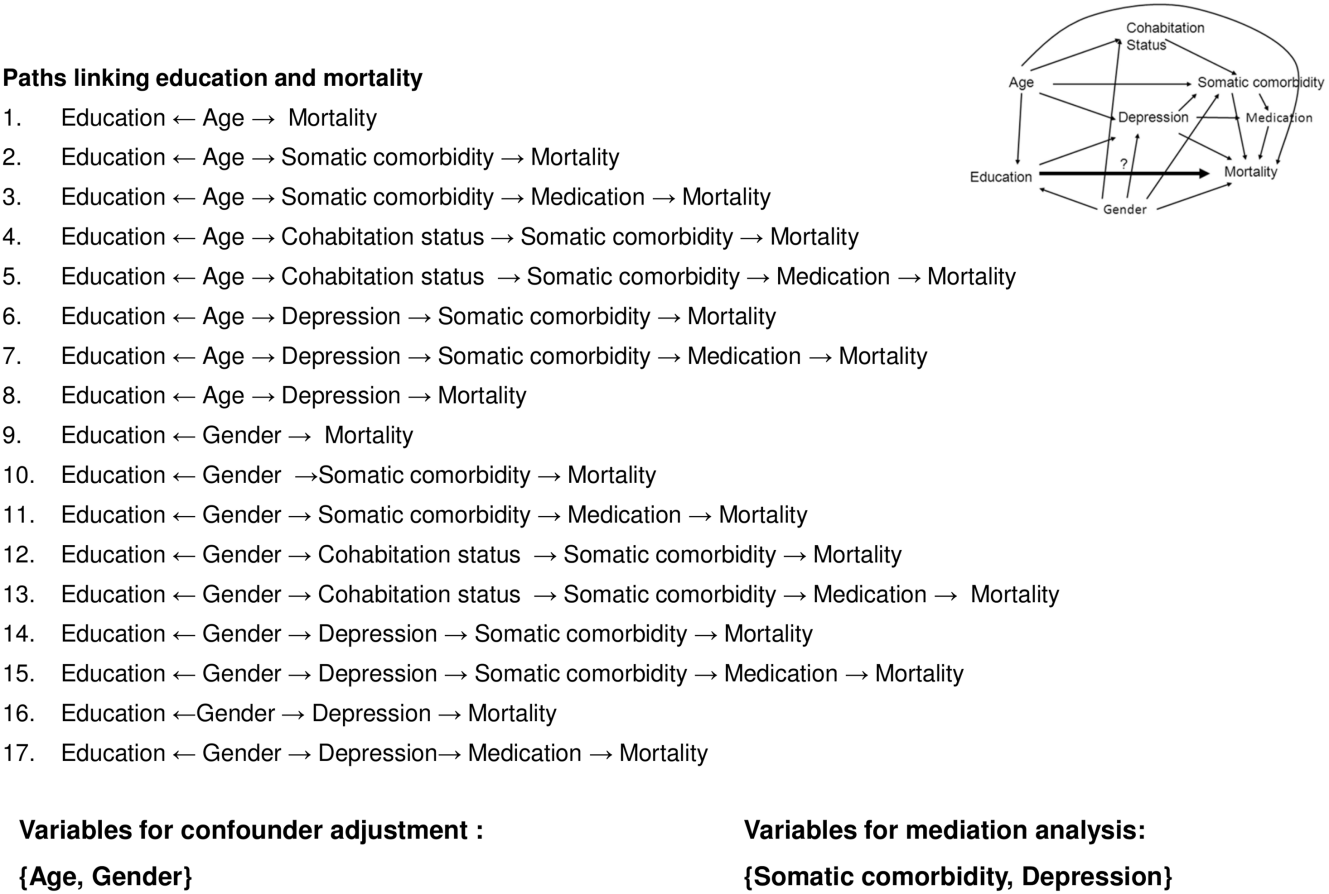
Directed acyclic graph of the assumed relationship between education and mortality with paths used for identifying potential confounding and mediating factors.

We analysed the association between educational status at study entry and mortality after 30 days, one year and at end of follow up using Cox regression with time since study entry as the underlying time scale. Person-years of follow-up were accumulated from time of study entry and were terminated at time of death, emigration, or the end of follow–up, whichever came first. Median follow-up was 5.0 years (range: 0.0–12.0 years). The covariables included in the multiple regression models were selected based on the directed acyclic graph [33] presented in Fig 1.

The inspection of the 17 paths linking education and mortality identified somatic comorbidity and depression as mediators and age and gender as confounding covariables. The influence of somatic comorbidities was examined both by entering the 9 disease item and as a sum score. Incident depression was entered as a time-dependent variable in the models. The proportional hazard assumption was examined by inspection of cumulative incidence plots and tested on the basis of Schoenfeld’s residuals. We examined whether the association between education and mortality was the same in ACS patients as in the reference by including an interaction term (Education*ACS patients/reference-status) tested using the likelihood ratio test. To explore if any educational differences in mortality were modified by comorbid depression, we introduced and tested the interaction term (Education*depression). All statistical analyses were carried out in STATA version 13.

## Results

Table 2 shows that in both ACS patients and in the reference population, subjects with low educational status had more somatic comorbidities and were more often statin users than the highly educated cohort members. Previous depression was also more frequent among the lower educated who also had higher rates of incident depressions. During 12 years follow-up, 29 583 (35.6%) of ACS patients and 19 105 (22.9%) of the reference population died. The crude death rates were highest among the lowest educated (Table 2).

**Table 2:** Characteristics of patients admitted first time with acute coronary syndrome (ACS) and a reference population in Denmark. According to educational status at study entry.

	ACS patients				Reference population*			
		Educational status				Educational status		
	N	Low	Middle	High	N	Low	Middle	High
All (Number)	83 062	39974	33417	9671	83 416	35134	34641	13641
**Baseline characteristics**								
***More than 2 comorbidities (%)***	56 736	42.2	31.2	8.8	44 170	37.8	31.3	11.4
***User of statins (%)***	20 351	24.5	24.7	23.6	10 820	13.8	13.0	10.7
Previous depression (%)	20 139	26.1	22.4	22.9	14 492	21.0	17.0	16.4
**Follow-up characteristics**								
Incident depression** (n)	16 571	8571	6248	1752	11 144	5563	4028	1553
Person year at risk for depression		55.0	51.0	15.0		58.9	61.6	24.4
***Depression rate/ 1000 py***		155.9	122.5	117.1		94.4	65.4	63.6
**Death**								
***Death 0–30 days after study entry (n)***	6676	3967	2128	581	1136	646	378	112
Person year at risk for death		3.0	2.6	0.8		2.8	2.8	1.1
***30 days mortality rate/ 1000 py***		1304.2	815.7	767.9		227.5	134.1	100.8
Death 0–1 year after study entry (n)	11 909	7079	3850	980	3032	1736	1015	281
Person year at risk for death		34.3	30.4	8.9		34.0	33.9	13.4
***1 year mortality rate/ 1000 py***		206.2	126.8	110.6		51.1	29.9	20.9
Death 0–12 year after study entry (n)	29 583	7328	9894	2361	19 105	10732	6441	1932
Person year at risk for death		214.9	199.9	59.2		225.9	234.7	93.2
***Mortality rate/ 1000 py***		80.6	49.5	39.9		47.5	27.4	20.7

* matched on gender, age, and municipality on time of ACS;

** depression diagnosed up to 2 years after study entry.

Due to the higher mortality in ACS patients, the absolute number of deaths, e.g. 1 year after study entry attributed to low education was 95 per 1000 py compared to 30 per 1000 py in the reference population. The Cox regression analyses showed a gradually increasing mortality rate during the first 30 days after study entry in the middle and lower educated compared to those with high education in both ACS patients and the reference population (Table 3).

**Table 3:** Association between educational status and all-cause mortality in patients admitted first time with acute coronary syndrome (ACS) and a reference population in Denmark.

	ACS patients		Reference population *	
	Hazard Rate Ratio (95%CI)		Hazard Rate Ratio (95% CI)	
Educational status	Adjusted**	Adjusted***	Adjusted**	Adjusted ***
	Mortality 30 days after study entry			
High	1	1	1	1
Middle	1.07(0.97–1.15)	1.06(0.98–1.16)	1.29(1.05–1.60)	1.26(1.03–1.55)
Low	1.27(1.16–1.39)	1.28(1.17–1.39)	1.44(1.18–1.77)	1.42(1.16–1.74)
	Mortality 1 year after study entry			
High	1	1	1	1
Middle	1.15 (1.08–1.23)	1.14(1.06–1.22)	1.40(1.22–1.59)	1.37(1.20–1.57)
Low	1.34(1.26–1.44)	1.34(1.23–1.41)	1.59(1.40–1.80)	1.56(1.37–1.77)
	Mortality at end of follow-up (median 5 years)			
High	1	1	1	1
Middle	1.26(1.20–1.32)	1.24(1.19–1.30)	1.29(1.22–1.35)	1.28(1.21–1.35)
Low	1.46(1.40–1.52)	1.45(1.39–1.51)	1.51(1.43–1.58)	1.48(1.41–1.56)

* matched on gender, age, and municipality on time of ACS;

**Adjusted for confounders: age and gender.

*** Adjusted for confounders: age and gender and mediating factors: somatic comorbidity, previous and incident depression.

Adjustment for the suggested confounding and mediating factors (Fig 1) had very slight influence on the associations. Similar associations with a dose-response pattern between educational level and mortality were seen for mortality after 1 year and during total follow-up. The tests for interactions also showed that the estimates for the influence of educational status on mortality did not differ between ACS patients and the reference population. Similar did comorbid depression not modify the relation between educational level and mortality. The sensitivity analyses based on the 13% of depression cases defined only by ICD-10 codes or with exclusion of the 10% with only a single prescription of antidepressants, gave results very similar to those presented in Table 3 based on depression defined by a discharge diagnosis or purchase of antidepressants independent of number of prescriptions purchased.

## Discussion

In agreement with a number of previous studies outlined in Table 1 we found that low educational status was associated with increased mortality after ACS. Although depression and somatic comorbidities were associated with education, these variables had limited impact on the association between education and mortality for both ACS patients and the reference population. As expected,low education was also associated with increased mortality in the reference population but to our surprise the relative mortality risks for lower educated were similar for ACS patients and the reference population. This indicates that the social inequality in survival after ACS is not a result of differences in the treatment of these patients but rather caused by some more common background factors, such as family environment [34].

The strengths of the present study are the large, unselected cohort of all patients admitted with first time ACS in the period from 2001 to 2009 in Denmark and the inclusion of a reference population. Importantly, however, subjects who experienced a fatal ACS event outside hospital as first manifestation of the disease were not included, but they form a minority of ACS patients with SEP inequalities similar to those for ACS patients dying in hospital [8,14]. Thus, we find it unlikely that this would influence the findings of the present study. Other strengths include valid information on educational status collected at the individual patient level, however, this information was missing for cohort members born before 1920 independent of their educational or disease risk. Consequently, we find it unlikely that it could induce selection bias. Information on previous and incident depression was classified based on hospital diagnoses and purchase of antidepressants from nation-wide registers with high completeness and good validity. The use of purchases of antidepressants in the definition of depression is a limitation due to risk of misclassification of cases that use antidepressants for other conditions and of the less severe depressions, which may be untreated or treated with psychotherapy. A random misclassification of this covariate might have reduced its explanatory impact. However, in sensitivity analysis based on the depression cases defined only by ICD-10 codes or when excluding those with only a single prescription of antidepressants, results were similar to those presented in this paper, thereby supporting the validity of the results.

In conclusion, the relative excess mortality rate in lower educated ACS patients seems to be comparable with the excess risk associated with low education in the background population and this inequality was not explained by somatic comorbidity or depression. Due to the higher mortality in ACS patients the absolute number of deaths attributed to low education was higher among these than in the reference population.
